# Author response to: Comment on Prophylactic mesh augmentation after laparotomy for elective and emergency surgery: meta-analysis

**DOI:** 10.1093/bjsopen/zrad130

**Published:** 2024-01-23

**Authors:** Simone Frassini, Paola Fugazzola, Lorenzo Cobianchi, Luca Ansaloni

**Affiliations:** University of Pavia, Pavia, Italy; Unit of General Surgery I, Fondazione I.R.C.C.S. Policlinico San Matteo, Pavia, Italy; University of Pavia, Pavia, Italy; Unit of General Surgery I, Fondazione I.R.C.C.S. Policlinico San Matteo, Pavia, Italy; University of Pavia, Pavia, Italy; Unit of General Surgery I, Fondazione I.R.C.C.S. Policlinico San Matteo, Pavia, Italy; University of Pavia, Pavia, Italy; Unit of General Surgery I, Fondazione I.R.C.C.S. Policlinico San Matteo, Pavia, Italy

Prophylactic mesh augmentation placement in the closure of midline laparotomy is a technique with promising clinical results, but at the same time, many surgeons remain concerned due to infections and surgical complications. Our group demonstrated in this recent systematic review and meta-analysis that prophylactic mesh placement has a significant decrease in incisional hernia (IH) rate at 1-, 2-, 3- and 4-year follow-up; at the same time, our results identified a trend of higher rates of wound dehiscence, wound complications and seroma but not reaching any statistical significance^1^.

We appreciate the correspondence by Rodriguez-Quintero and Romero-Velez, especially because the authors underline some unclear and debated topics. The authors claim a legal aspect and ‘fear of litigation’ have a role in the surgical decision-making process at the moment of laparotomy closure technique.

In our analysis, there were no clear and robust data on technical indications in elective settings, but our point of interest was on high-risk patients in emergency settings. According to subgroup analysis, at 1- and 2-year follow-up comparing elective and emergency surgery, the IH rate in the emergency group was even lower than the comparison elective group. In addition, recent ECLAPTE guidelines by the World Society of Emergency Surgery stated that prophylactic mesh augmentation appears to be effective in preventing IH and is safe for postoperative complications when treating patients with increased risk of IH development. Therefore, the speculative barrier related to the legal aspect could be less incisive in emergency settings.

Prophylactic mesh placement should be considered a promising technique for preventing IH after midline laparotomy closure, and the application considered where available and safe. Future perspectives are needed to support surgical decisions even with the ‘fear of litigation’.
